# Preclinical evaluation of [^18^F]FDG-PET as a biomarker of lymphoid tissue disease and inflammation in Zika virus infection

**DOI:** 10.1007/s00259-022-05892-9

**Published:** 2022-07-25

**Authors:** Carla Bianca Luena Victorio, Joanne Ong, Jing Yang Tham, Marie Jennifer Reolo, Wisna Novera, Rasha Msallam, Satoru Watanabe, Shirin Kalimuddin, Jenny G. Low, Subhash G. Vasudevan, Ann-Marie Chacko

**Affiliations:** 1 Laboratory for Translational and Molecular Imaging, Cancer and Stem Cell Biology Programme, Duke-NUS Medical School, 8 College Road, Singapore, 169857 Singapore; 2 Programme in Emerging Infectious Disease, Duke-NUS Medical School, 8 College Road, Singapore, 169857, Singapore; 3 Department of Infectious Diseases, Singapore General Hospital, 20 College Road, Singapore, 169856 Singapore

**Keywords:** [^18^F]FDG, Zika, Viral infection, Dengue, AG129 mouse, Viral inflammation

## Abstract

**Purpose:**

Zika (ZIKV) is a viral inflammatory disease affecting adults, children, and developing fetuses. It is endemic to tropical and sub-tropical countries, resulting in half the global population at risk of infection. Despite this, there are no approved therapies or vaccines against ZIKV disease. Non-invasive imaging biomarkers are potentially valuable tools for studying viral pathogenesis, prognosticating host response to disease, and evaluating in vivo efficacy of experimental therapeutic interventions. In this study, we evaluated [^18^F]fluorodeoxyglucose ([^18^F]FDG)-positron emission tomography (PET) as an imaging biomarker of ZIKV disease in a mouse model and correlated metabolic tracer tissue uptake with real-time biochemical, virological, and inflammatory features of tissue infection.

**Methods:**

[^18^F]FDG-PET/CT imaging was performed in an acute, lethal ZIKV mouse infection model, at increasing stages of disease severity. [^18^F]FDG-PET findings were corroborated with ex vivo wholemount-tissue autoradiography and tracer biodistribution studies. Tracer uptake was also correlated with in situ tissue disease status, including viral burden and inflammatory response. Immune profiling of the spleen by flow cytometry was performed to identify the immune cell subsets driving tissue pathology and enhancing tracer uptake in ZIKV disease.

**Results:**

Foci of increased [^18^F]FDG uptake were consistently detected in lymphoid tissues—particularly the spleen—of ZIKV-infected animals. Splenic uptake increased with disease severity, and corroborated findings in tissue pathology. Increased splenic uptake also correlated with increased viral replication and elevated expression of pro-inflammatory cytokines within these tissues. ZIKV-infected spleens were characterized by increased infiltration of myeloid cells, as well as increased proliferation of both myeloid and lymphoid cells. The increased cell proliferation correlated with increased tracer uptake in the spleen. Our findings support the use of [^18^F]FDG as an imaging biomarker to detect and track ZIKV disease in real time and highlight the dependency of affected tissue on the nature of the viral infection.

**Conclusion:**

[^18^F]FDG uptake in the spleen is a useful surrogate for interrogating in situ tissue viral burden and inflammation status in this ZIKV murine model.

**Supplementary Information:**

The online version contains supplementary material available at 10.1007/s00259-022-05892-9.

## Introduction

Zika virus (ZIKV) is a mosquito-transmitted infection affecting developing fetuses, children, and adults. It causes sporadic outbreaks, the largest of which occurred in 2015 and was declared a public health emergency of international concern by the World Health Organization (WHO) [1]. Half of the global population is currently at risk of infection and live in tropical and subtropical regions where *Aedes* mosquitoes—the vector responsible for the transmission of ZIKV—are endemic [2, 3]. With global climate change, rapid population growth in the tropics, and an expected return to high-volume air travel in the post-COVID pandemic era, even more people will be at risk of exposure to such mosquito-borne viruses. Despite the continued public health threat of ZIKV, no therapeutics or vaccines are approved for human use. Various vaccine candidates and antivirals were developed and evaluated in clinical trials (reviewed in [4, 5]) but met with various challenges in recent years. Vaccine and therapeutic efficacy trials are hampered by the difficulty in patient recruitment due to the low natural ZIKV infection rates and require controlled deliberate infection of trial participants [6]. Moreover, global efforts in vaccine development have been dampened by refocusing of resources towards the current COVID pandemic.

One way to expedite the bench-to-bedside development of these therapies is by employing non-invasive imaging biomarkers. These facilitate spatial localization of viral disease (or disease severity) and enable deeper in situ molecular insights into disease pathogenesis. This is not attainable with the current diagnostic gold standard of detecting active viral replication in peripheral blood using polymerase chain reaction (PCR) [7]. Imaging biomarkers that correlate with disease progression have the potential to predict response to therapy and rapidly inform go or no-go decisions in drug evaluations. This is most relevant in the context of developing therapeutics against highly virulent and lethal pathogens—such as Ebola and Nipah viruses—but is nonetheless relevant to ZIKV and other related flavivirus diseases, including the endemic dengue virus (DENV) [8]. Therefore, there is a real clinical need to identify and characterize infectious disease-related imaging biomarkers [9].

We previously identified ^18^F-fluorodeoxyglucose ([^18^F]FDG) uptake as a preclinical imaging biomarker for early prediction of response to therapeutic interventions for DENV infection. Lesions of increased [^18^F]FDG uptake in the intestines were found to correlate with disease severity, tissue viral burden, and expression of inflammatory cytokines in lethal and non-lethal murine models of DENV infection [10]. Clinical [^18^F]FDG-PET also distinguishes infection-mediated tissue inflammation patterns, namely, in lymphoid tissues, in DENV patients during early disease [11, 12]. In contrast, there have been no reports of [^18^F]FDG-PET imaging in ZIKV disease, in either humans or animal models.

Here we examined [^18^F]FDG uptake in the lymphoid organs of a murine model of acute lethal ZIKV disease. The spatial and temporal dynamics of metabolic tracer uptake in ZIKV were also compared with DENV to identify common tissue-specific imaging biomarkers between these two related viruses. Tissue uptake in ZIKV was further correlated with pathology and markers of viral disease and inflammation in tissues. This study demonstrates that splenic [^18^F]FDG uptake is a robust surrogate for interrogating of tissue viral burden and inflammation status in ZIKV.

## Methods

### Study ethics

Animal experiments (protocol 2016/SHS/1197) were performed with approval from the Institutional Animal Care and Use Committee (IACUC) of Singapore Health Services and conformed to the US National Institutes of Health (NIH) guidelines and public law.

### Animal infection models

Male AG129 mice, deficient in expression of receptors for type I and type II interferons, were obtained from an in-house breeding colony, and subsequently used for infection studies at 8–11 weeks of age. This transgenic mouse model is the classic murine model for flavivirus infection, as wild-type mouse strains that normally express type I/II interferons are not susceptible to ZIKV and DENV infections [13, 14] Moreover, the potent antiviral effects of type I/II interferons significantly dampen the infection, replication, and dissemination of these viruses [15, 16]. Lethal infection models were generated using either a clinical ZIKV strain Paraiba01/2015 (GenBank Accession No. KX280026.1) isolated from an anonymous patient in the state of Paraiba, Brazil (a kind gift from Dr. Pedro Vasconcelos (Instituto Evandro Chagas, Brazil)), or using mouse-adapted DENV2 strain S221 [17] (a gift from Prof. Sujan Shresta (La Jolla Institute for Allergy and Immunology, USA)).

The ZIKV mouse model was established by intraperitoneal (i.p.) injection of 10^5^ pfu of ZIKV as described previously [18–20]. Mouse infections with DENV were performed as previously described [10, 19]. Briefly, mice were injected intravenously (i.v.) with DENV2 S221 (5 × 10^4^ pfu) complexed with 4G2 mouse monoclonal antibody (mAb; 20 μg) that targets the flavivirus envelope protein.

**Fig. 1:**
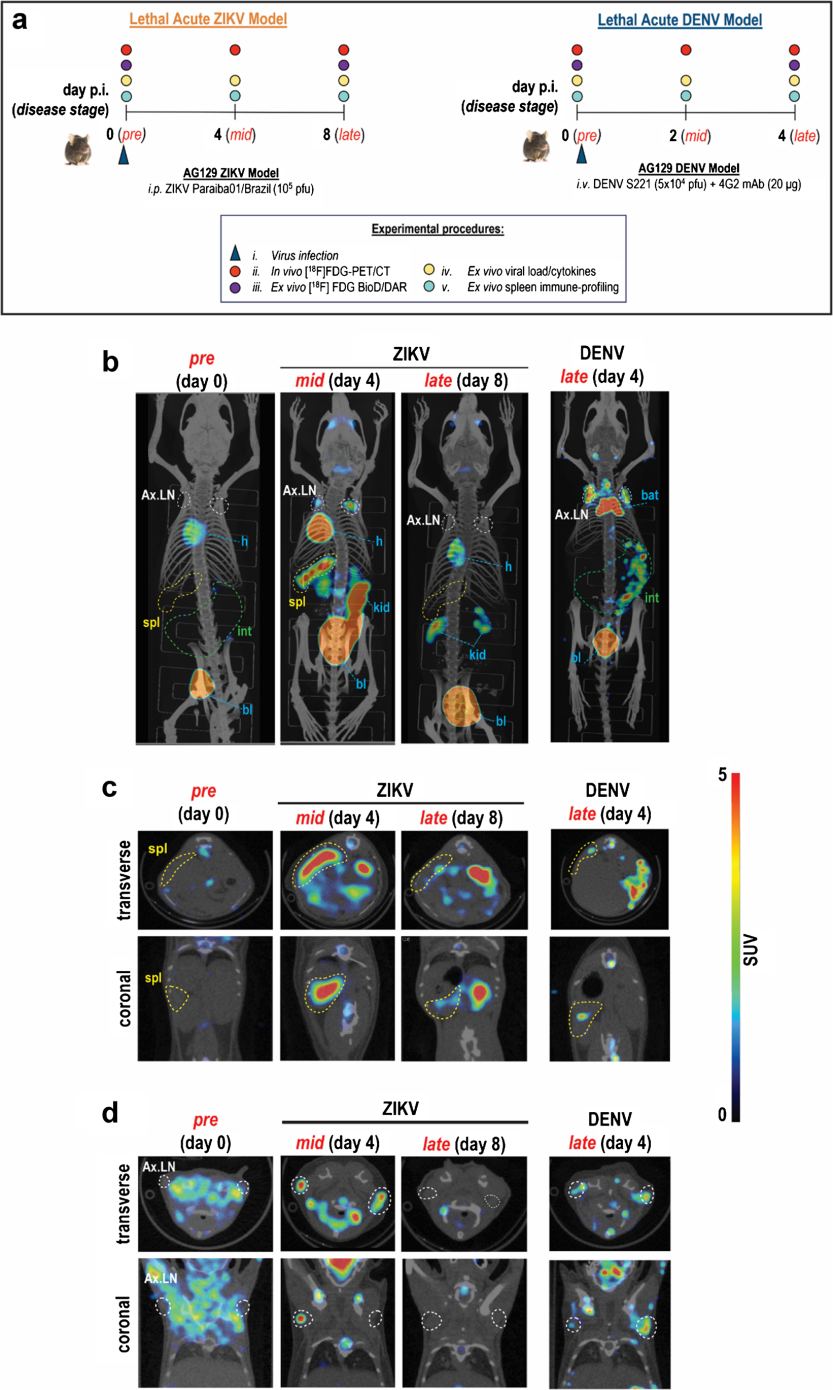
Total-body [^18^F]FDG uptake in acute ZIKV infection compared to DENV infection. **a** Study timeline and experimental procedures for the lethal acute Zika virus (ZIKV) and dengue virus (DENV) infection models. Detailed experimental procedures are described in the legend. Disease stages in each model are shown with corresponding days post-infection in the timeline. *pre*, *pre*-infection; *mid*, mid-disease; *late*, late disease. **b** Representative maximum intensity projections (MIP) of total-body [^18^F]FDG-PET/CT in ZIKV-infected (*n* = 10) and DENV-infected (*n* = 6) mice. Images were acquired 60 min post tracer injection. **c**, **d** Representative PET/CT image slices highlighting [^18^F]FDG uptake in **c** spleens and **d** axillary lymph nodes of infected mice. Tissue boundaries are shown in dashed lines. *p.i.*, post-infection. *pfu*, plaque-forming units. *i.p.*, intra-peritoneal injection. *i.v.*, intra-venous injection. *Ax.LN*, axillary lymph node. *spl*, spleen. *h*, heart. *kid*, kidneys. *bat,* brown adipose tissue. *bl*, bladder. *int*, intestines

The study timelines and selection of disease stages for experimental procedures are summarized in Fig. 1a. The time points for the assays were selected based on the Kaplan–Meier survival curves and body weight monitoring of each disease model (Fig. S1a, b). *Late disease* was designated as the time point when the animals started dying from disease—*i.e.*, day 8 for the ZIKV model and day 4 for the DENV model (Fig. S1a). Meanwhile, *mid disease* was designated as the midpoint between *pre disease* and *late disease* and also coincides with when the animals began losing significant weight (Fig. S1b).

### [^18^F]FDG-PET/CT image acquisition, image reconstruction, and image analysis

[^18^F]FDG-PET/CT imaging in mice was performed as described previously [10]. Briefly, mice were fasted overnight (~ 12 h), sedated with 2% isoflurane, and i.v. injected with 40 MBq [^18^F]FDG (Singapore Radiopharmaceuticals, Singapore). Following a 1-h uptake phase, mice underwent total body PET scan for 40 min (VECTOR^4^CT, MILabs, Netherlands) using the M7 UHR 0.7-mm pinhole collimator, immediately followed by total body accurate CT scan for 5 min (tube current, 0.24 mA; tube voltage, 50 kV). The resulting images were reconstructed at a voxel resolution of 0.8 mm using a SROSEM algorithm with 8 subsets, 12 iterations, and a 1.6-μm Gaussian filter. The PET and CT image co-registration was automatically handled by the MILabs image reconstruction software package. Similarly, attenuation correction of the PET image was done automatically with the software using the CT image.

Image analysis was performed using PMOD v3.7 (PMOD Technologies, Switzerland). Volumes of interest (VOIs) were drawn on CT images for lymph nodes and guts and transferred to PET images. Spleen VOIs were drawn using both CT and PET images and subjected to iso-contouring using a minimum threshold of SUV = 1.0—the count rate when a given activity is uniformly distributed within a body phantom (or VOI). Gut VOIs were drawn around the abdominal cavity using CT as a guide and excluding the PET signals from both kidneys and spleen. The mean standardized uptake value (SUV) was defined as the average of all voxels in the VOI.

### Ex vivo digital autoradiography and biodistribution of [^18^F]FDG uptake in virus-infected tissues

Infected mice were injected i.v. with 10 MBq [^18^F]FDG and tissues were harvested following a 60-min tracer uptake. Freshly isolated wholemount lymphoid tissues were immediately exposed to multi-purpose phosphoscreen (BAS-IP MS) for 30 min. Fresh tissues with high tracer uptake were exposed to super-resolution (BAS-IP SR) phosphoscreen for 5 min (GE Healthcare Life Sciences, USA). [^18^F]FDG standards at 2/3 serial dilution from 600 to 0 kBq were mounted together with mouse tissue for calibration of digital autoradiography (DAR) images. Screens were then scanned using the Sapphire Biomolecular Imager (Azure Biosystems, USA) at 100-μm resolution. Images were processed and analyzed using ImageJ software (National Institutes of Health, USA). Following DAR, tissues were subjected to gamma counting (2470 Wizard2, PerkinElmer, USA).

### Tissue viral loads and pro-inflammatory cytokine expression

Tissues were harvested immediately after PET/CT imaging and stored in − 80 °C. Frozen tissues were homogenized, and viral RNA was extracted using QIAamp Viral RNA Mini Kit (Qiagen, Germany). Real-time qRT-PCR was carried out using primers and protocols as described previously [19]. Cytokine expression was also measured from homogenized frozen tissues using Ready-Set-Go! ELISA kits for murine TNF-α and IL-6 and following the manufacturer’s recommended protocols (eBioscience, USA).

### Immune profiling of spleen tissues

Tissue immune profiling was conducted as previously described [21, 22]. Briefly, spleens were harvested, mechanically disaggregated, and incubated in digestion buffer containing DNaseI and collagenase. Following homogenization, single-cell suspensions of splenocytes were collected by passing the suspension through a nylon mesh strainer (70 μm). Cells were labeled with fluorescent antibody cocktails, and immune cell subsets were gated and identified by flow cytometry (Fortessa, BD Biosciences, USA) and analyzed using FlowJo V10.8.0 (Becton Dickinson & Company (BD), USA). Detected events were normalized to absolute cell number using CountBright Absolute Counting Beads (Thermo Fisher, USA) [23]. The following markers were used: DAPI^−^ (live cells), CD45^+^ (total hematopoietic cells or immune cells), Ly6G^+^Ly6C^+^CD11b^+^ (granulocytes); CD11b^+^F4/80^+^Ly6c^+^Ly6G^−^CD3^−^ (monocyte-derived macrophages), CD11b^+^CD115^+^ (total monocytes), CD11c^+^MHCII^+^ (conventional dendritic cells), CD3^−^﻿CD19^+^B220^+^MHCII^+^ (B cells), CD19^−^CD49b^−^B220^−^LY6G^−^CD3^+^ (total T cells), CD19^−^CD49b^−^B220^−^LY6G^−^CD3^+^CD8^+^ (cytotoxic T cells), and CD19^−^CD49b^−^B220^−^LY6G^−^CD3^+^CD4^+^ (Helper T cells).

### Statistical analysis

Statistical analyses and graphing were performed with Prism v8.0 (GraphPad Software, USA). Non-parametric Kruskal–Wallis test with Dunn’s post-hoc correction was used to compare means, and Spearman’s coefficient (*ρ*) was used to assess the correlation between tissue [^18^F]FDG uptake and various parameters. The best-fit linear regression trend of the scatter plots and 95% confidence interval (CI) for the regression line were also computed. Results were considered statistically significant at *p* < 0.05.

## Results

### [^18^F]FDG-PET/CT imaging in acute and lethal ZIKV

We infected AG129 mice with ZIKV and injected the animals with [^18^F]FDG to investigate tissue-specific changes in tracer uptake that could be correlated with various aspects of disease progression. The study schedule was optimized to capture disease- and host response-related biomarker dynamics at increasing stages of disease severity (Fig. 1a). PET/CT was performed at pre-infection (day 0), mid-disease (day 4), and late-disease (day 8) in the ZIKV model (Fig. 1a). In the comparable DENV model [10], mid and late diseases were reflected on days 2 and 4 post-infection, respectively. We compared [^18^F]FDG uptake in ZIKV to our previously published [^18^F]FDG-PET/CT findings in DENV [10], re-analyzed with respect to lymphoid tissues.

**Fig. 2:**
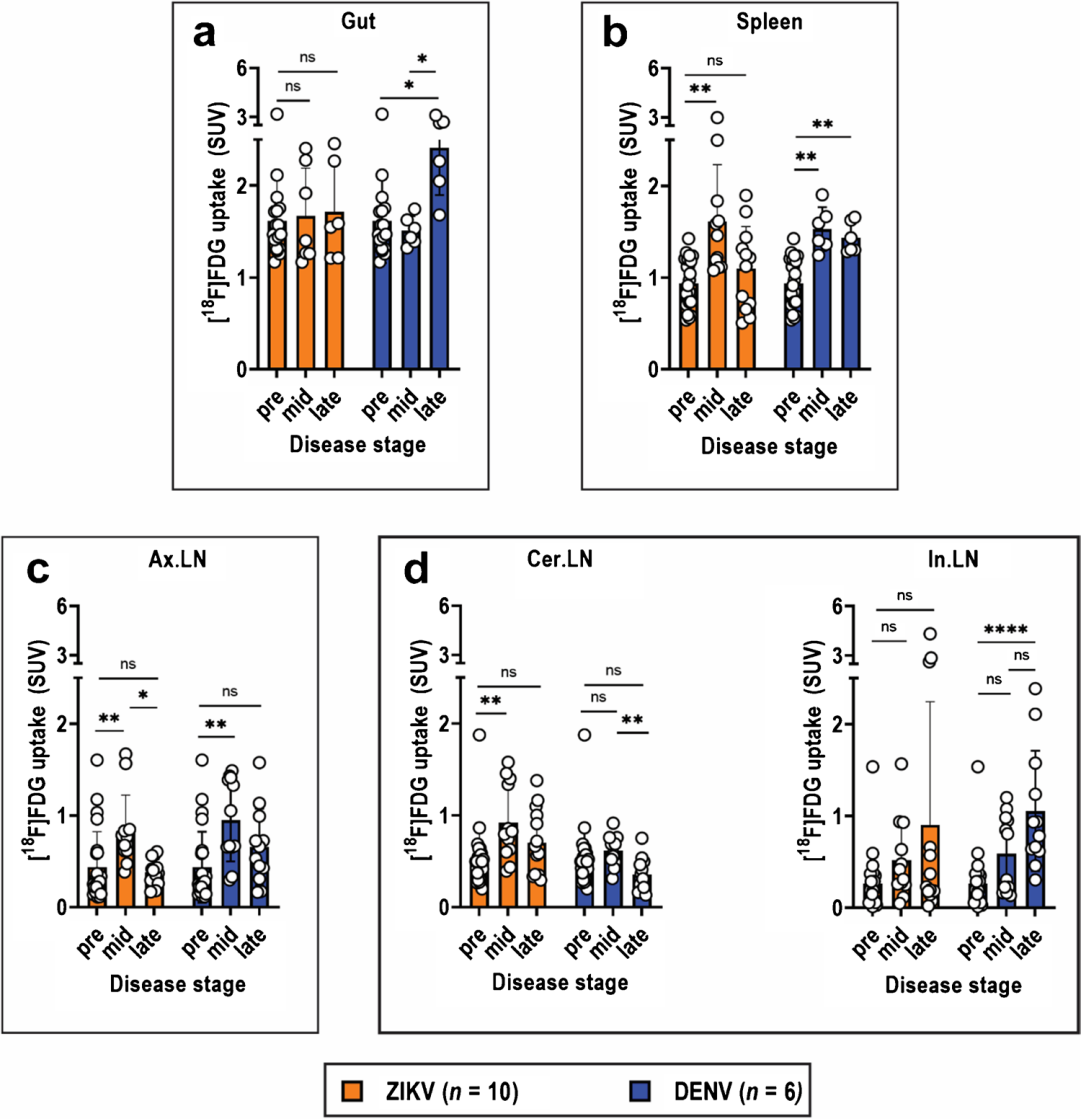
Quantification of in vivo [^18^F]FDG uptake from PET images. Mean SUV quantification of [^18^F]FDG uptake in VOIs (volumes of interest) drawn around the **a** gut, **b** spleen, **c** axillary lymph nodes (Ax.LN), **d** cervical lymph nodes (Cer.LN), and inguinal lymph nodes (In.LN). VOIs were drawn on PET/CT images acquired at pre-infection (pre; day 0), mid (day 4), and late (day 8) ZIKV disease. For DENV, these time points correspond to days 0, 2, and 4, respectively. Data from ZIKV mice (*n* = 10) and DENV mice (*n* = 6) at each disease stage are presented as mean ± SD, and individual points represent data from each mouse. Data for pre-infection (*n* = 16) are pooled from pre-infection data from both ZIKV and DENV models. Multiple comparison of means was performed using Kruskal–Wallis test with Dunn’s post hoc correction. *p*-values are represented in different symbols *p < 0.05. **p < 0.005. ****p < 0.0001. *ns*, not significant

The salient feature in the ZIKV [^18^F]FDG-PET/CT maximum intensity projection (MIP) images was the increased tracer uptake in lymphoid tissues, particularly the spleen (spl) and axillary lymph nodes (Ax.LN), at mid disease (day 4 post-infection) (Fig. 1b-d). No detectable signal was observable in the bone marrow (*i.e.*, femur and spine). The most striking organ-specific tracer uptake in the DENV model was from the gut (int), which increased over time until the animal succumbed to disease (Figs. 1b  and 2a). However, no changes in tracer uptake were observed in the gut at progressive stages of ZIKV disease (Figs. 1b and 2a).

[^18^F]FDG ZIKV spleen uptake peaked mid-disease on day 4 (SUV 1.6) and was 72% higher than pre-infection at day 0 (SUV 0.9; *p* = 0.004) (Fig. 2b; Table S1). Similarly, tracer uptake in DENV spleens peaked at mid disease on day 2 (SUV 1.5) and was 63% higher than pre-infection (SUV 0.9; *p* = 0.001) (Fig. 2b; Table S1). Both models also exhibited increased uptake in Ax.LN that peaked at mid-disease relative to pre-infection. Uptake in ZIKV Ax.LN on day 4 (SUV 0.8) was 90% higher than at day 0 (SUV 0.4, *p* = 0.0005) (Fig. 2c; Table S1), while uptake in DENV Ax.LN on day 2 (SUV 0.9) was 118% higher than day 0 (SUV 0.4, *p* = 0.002). Notable increases in tracer uptake was also observed in ZIKV cervical lymph nodes (Cer.LN) during mid-disease but not inguinal lymph nodes (In.LN) at any stage of disease (Fig. 2d; Table S1). These results are in contrast to DENV, where increased tracer uptake in In.LN was observed at late disease (day 4) and no change in Cer.LN at any disease stage (Fig. 2d; Table S1).

### Ex vivo DAR and biodistribution of [^18^F]FDG in infection models

**Fig. 3:**
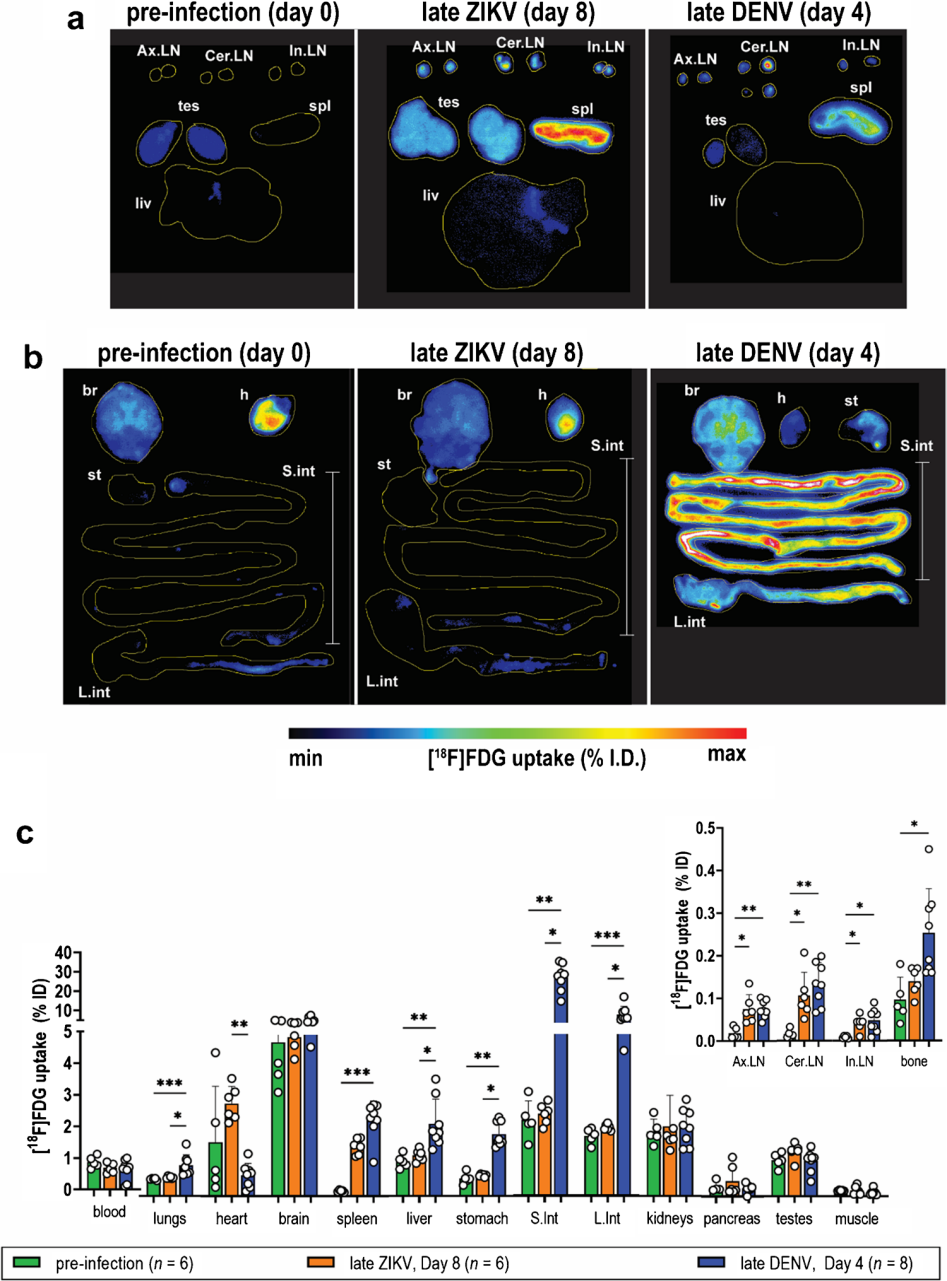
Ex vivo assessments of tissue [^18^F]FDG uptake in acute ZIKV and DENV disease. **a, b** Representative wholemount tissue ex vivo digital autoradiography (DAR) images of **a** lymphoid tissues, testes, and liver and **b** brain, heart, and digestive tract from pre-infection (*n* = 5), late ZIKV (*n* = 6), and late DENV (*n* = 8) mice. **c** [^18^F]FDG biodistribution in various mouse tissues measured by gamma counting*.* Tissues from late ZIKV mice (*n* = 6) at day 8 post-infection and late DENV mice (*n* = 8) at day 4 post-infection were harvested at 60 min after tracer injection and subjected to DAR and gamma counting. *Ax.LN,* axillary lymph nodes. *Cer.LN*, cervical lymph nodes. *In.LN*, inguinal lymph nodes. *tes*, testes. *spl*, spleen. *liv*, liver. *br*, brain. *h*, heart. *st*, stomach. *S.int*, small intestines. *L.int*, large intestines. Values are shown as mean ± SD. Multiple comparison of means was performed using Kruskal–Wallis test with Dunn’s post hoc correction. *p*-values are represented in different symbols. **p < 0.005. ***p < 0.0005

Increased [^18^F]FDG uptake in lymphoid tissues was also observed by ex vivo tissue wholemount DAR images, which revealed elevated tracer uptake in spleens (spl) and lymph nodes (Ax.LN, Cer.LN, In.LN) of both ZIKV and DENV mice at late disease (Fig. 3a). Expectedly, hotspots of high tracer uptake were also detected in metabolically active tissues—brain (br) and heart (h)—in both models (Fig. 3b). Moreover, no uptake was detected in the stomach (st) and intestines (S.Int, L.Int) from ZIKV animals, which was in stark contrast to DENV (Fig. 3b). These uptake patterns are consistent with the observed changes in gross pathology (Fig. S1c, d). Elevated uptake was also observed in some ZIKV-infected testes (tes) (Fig. 3a), which are tissue targets for ZIKV replication. These signals were obscured by the bladder in PET/CT images.

[^18^F]FDG biodistribution was also evaluated from the same tissues used for DAR imaging. Tissues were harvested at late disease, on day 8 post-infection for ZIKV and day 4 post-infection for DENV (Fig. 1a). No difference in uptake was observed in ZIKV-infected testes (Fig. 3c) despite observations in DAR. Meanwhile, the increased tracer distribution in the digestive tissues of DENV mice, and its lack thereof in ZIKV mice, was consistent with observations from both PET and DAR (Figs. 1b and 3b, c). More relevant to this study is the increased uptake in lymphoid tissues in both ZIKV and DENV disease. Notably, [^18^F]FDG in ZIKV spleens was 879% higher than pre-infection spleens (1.54 vs 0.16% ID, respectively; *p* = 0.0001), while uptake in DENV spleens was 1400% higher than uptake in pre-infection spleens (2.37% ID, *p* = 0.0007) (Fig. 3c; Table S2). All lymph nodes collected also registered higher uptake in disease as compared to pre-infection control. FDG activity in ZIKV Ax.LN was 305% higher than activity detected in pre-infection (0.07 vs. 0.02% ID, *p* = 0.03), while the amount of tracer detected in DENV Ax.LN (0.08%ID) was 306% higher than in the control group (*p* = 0.005) (Fig. 3c; Table S2). In general, tissue biodistribution corroborated the findings from PET imaging, particularly in the intestines, spleen, and lymph nodes. However, one difference was observed in the bone marrow. PET imaging could not distinguish changes in ZIKV bone uptake in the spine or other long bones in infected mice, and this was confirmed with biodistribution studies. In contrast, a significant increase in [^18^F]FDG uptake in femur bones was only observed by gamma counting in DENV mice at late disease relative to pre-infection (0.25 vs. 0.10% ID; *p* = 0.01) (Fig. 3c; Table S2). However, it is unclear whether the bone uptake is due to free ^18^F-fluoride in the bone or due to a possible lymphoid inflammatory response within the bone marrow.

### Correlation of [^18^F]FDG PET and ZIKV disease status in lymphoid tissues

**Fig. 4:**
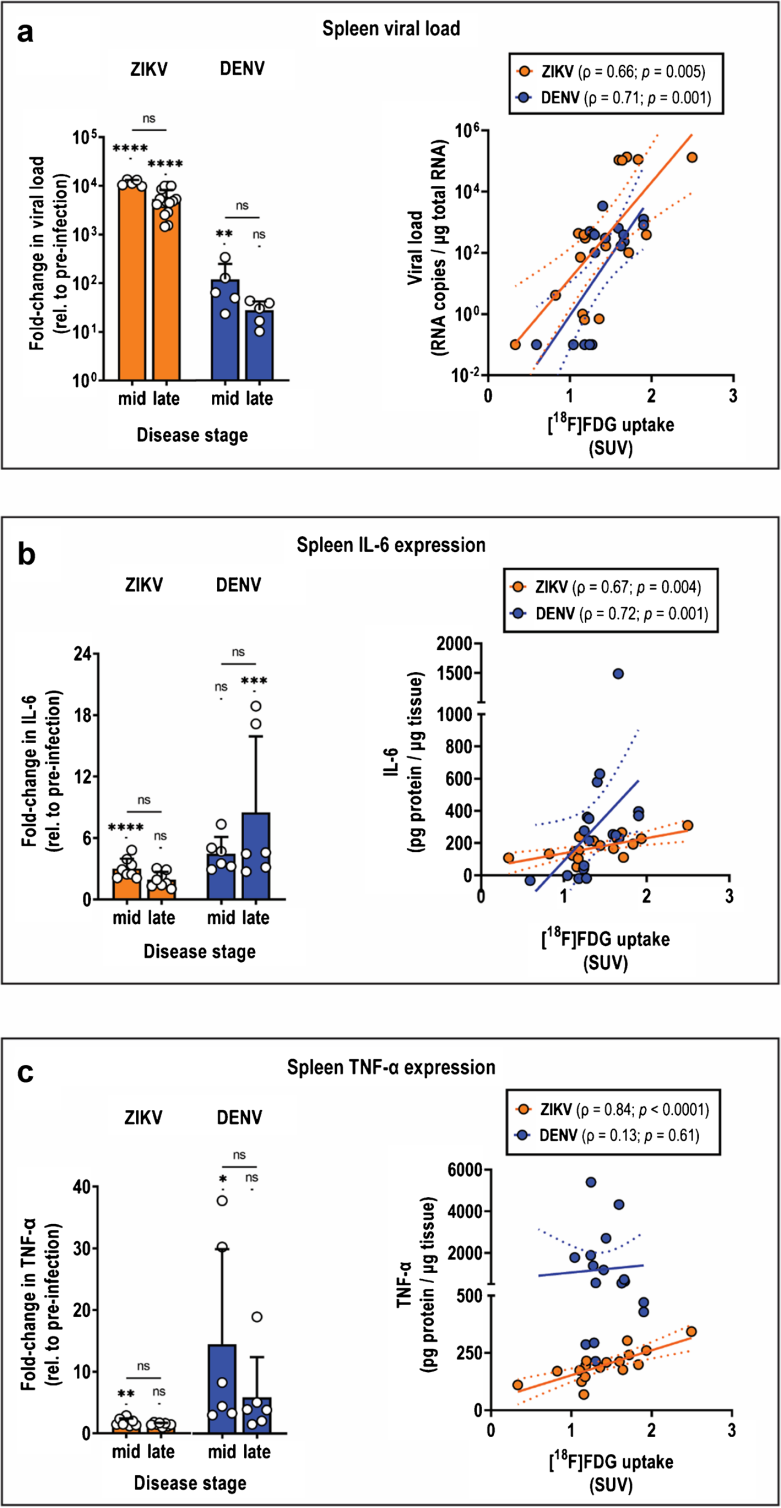
Contribution of tissue viral burden and inflammation status on spleen [^18^F]FDG uptake in acute ZIKV and DENV disease. **a**, **c** Fold change in expression at mid and late disease relative to pre-infection, and correlation between [^18^F]FDG uptake and either **a** tissue viral load, **b** IL-6 expression, or **c** TNF-α expression in diseased spleens. Spleens from two independent experiments were harvested from ZIKV mice at mid disease (*n* = 10) and late disease (*n* = 10), and DENV mice at mid disease (*n* = 5) and late disease (*n* = 5). Spleens from pre-infection mice (*n* = 10) were also obtained from two independent experiments. Data are presented as mean ± SD. Multiple comparison of means was performed using Kruskal–Wallis test with Dunn’s post-hoc correction. *p < 0.05. **p < 0.005. ***p < 0.0005. ****p < 0.0001. *ns*, not significant. Solid lines indicate the best-fit linear regression trend of the scatter plots, and dotted lines indicate the 95% CI for the regression line. ρ, Spearman correlation coefficient

Active viral replication is a principal contributor to pathology and ultimately death in preclinical models. Hence, we compared [^18^F]FDG SUV to ZIKV viral burden in tissues. In ZIKV spleen, viral burden at mid disease and late disease was 11,500-fold higher (*p* < 0.0001) and 5000-fold higher (*p* < 0.0001) than at pre-infection, respectively, and strongly correlated with tracer uptake (*ρ* = 0.66; *p* = 0.007) (Fig. 4a). In addition, despite viral burden in DENV spleen being 100–200-fold less than in ZIKV spleen, viral load also strongly correlated with tissue uptake in DENV (*ρ* = 0.71; *p* = 0.001) (Fig. 4a). These indicate that [^18^F]FDG uptake in the spleen is a sensitive indicator of viral burden in ZIKV, as well as in DENV.

Tissue inflammation is associated with ZIKV disease, particularly within the brain, gonads, and eyes, as observed in various preclinical models. Expression of pro-inflammatory cytokines interleukin-6 (IL-6) and tumor necrosis factor-α (TNF-α) in ZIKV spleen at both mid and late disease was two-fold to three-fold higher than pre-infection (Fig. 4b,c). Additionally, both cytokines showed a strong correlation with [^18^F]FDG uptake (IL-6: *ρ* = 0.67; CI: 0.27–0.88; *p* = 0.004; TNF-α: *ρ* = 0.84; CI: 0.59–0.94; *p* < 0.0001), confirming that uptake in ZIKV spleens was also a sensitive indicator of tissue inflammation status. This contrasted with DENV, where only IL-6 exhibited a strong correlation with [^18^F]FDG PET signals in the spleen (*ρ* = 0.72; CI: 0.38–0.89; *p* = 0.0007), despite IL-6 and TNF-α having more exaggerated expression in DENV spleens relative to ZIKV (8–14-fold higher than pre-infection) (Fig. 4b,c).

Of the other lymphoid tissues, Cer.LN and In.LN were not evaluated due to lack of strongly significant changes in [^18^F]FDG SUV in ZIKV. In ZIKV Ax.LN, viral burden significantly increased at mid and late disease compared to pre-infection (*p* < 0.0001) (Fig. S2a), and exhibited a strong positive correlation with tissue tracer uptake (*ρ* = 0.75; *p* = 0.001) (Fig. S2b). However, expression of both IL-6 and TNF-α declined over the course of disease and did not exhibit any correlation with tissue uptake (Fig. S2a, b). These results indicate that [^18^F]FDG uptake in ZIKV Ax.LN can only be used as an indicator of tissue viral replication but not of inflammation status.

### Immune profiling of ZIKV virus-infected spleens

**Fig. 5:**
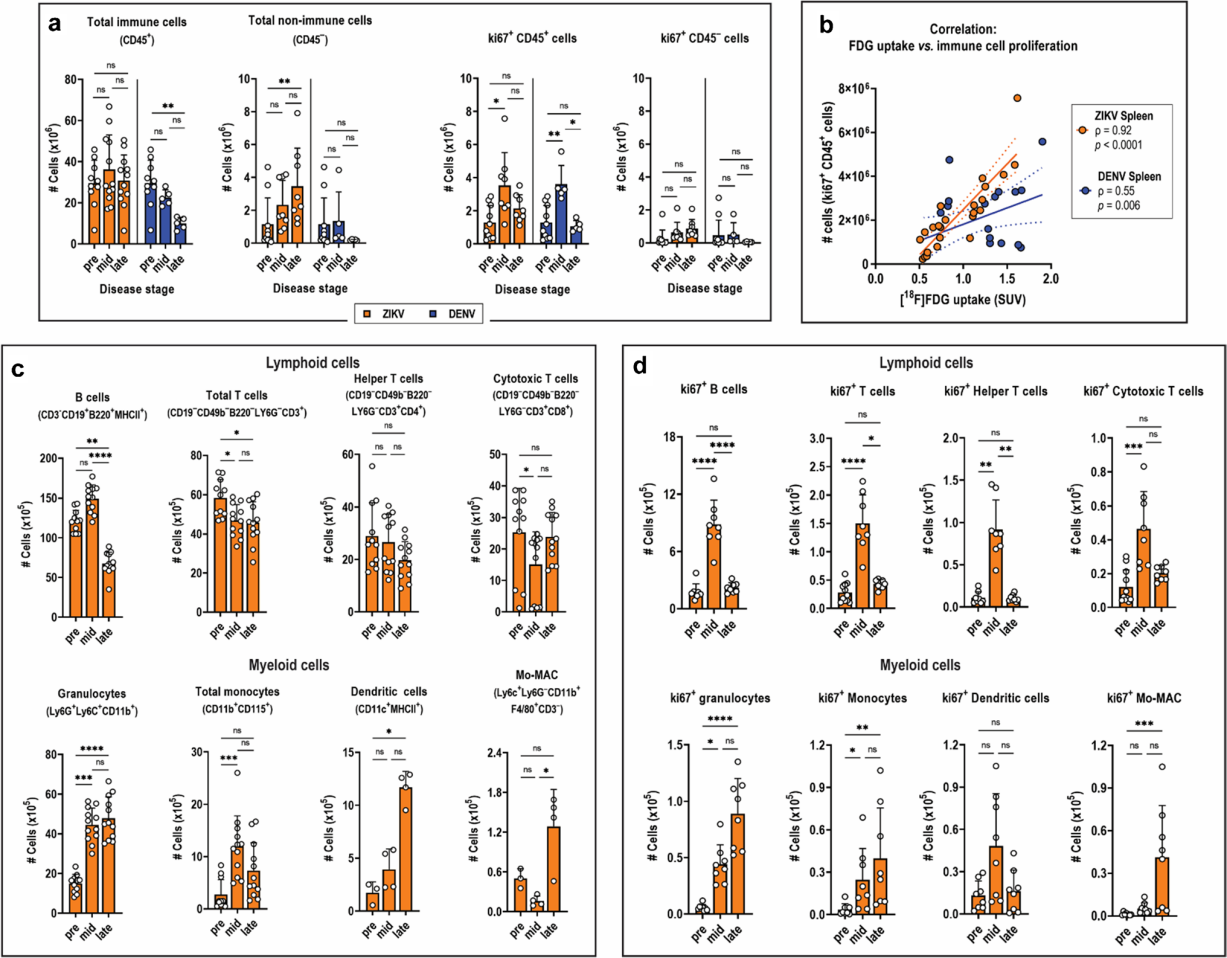
Immune cell profile and proliferation in ZIKV infected spleens. **a** Quantification of absolute cell numbers of proliferating (ki67^+^) splenocytes/immune cells (CD45^+^ cells) and non-immune cells (CD45^¯^ cells). **b** Correlation between ki67 expression in CD45^+^ immune cells and [^18^F]FDG uptake in the spleen. Solid lines indicate the best-fit linear regression trend of the scatter plots, and dotted lines indicate the 95% CI for the regression line. ρ, Spearman correlation coefficient. **c**, **d** Quantification of absolute numbers of **c** various lymphoid and myeloid immune cell subsets, and **d** proliferating (ki67^+^) lymphoid and myeloid cells in ZIKV spleens. Spleens were harvested from ZIKV-infected mice at mid disease (day 4; *n* = 12) and late disease (day 8; *n* = 8); from DENV-infected mice at mid disease (day 2; *n* = 5) and late disease (day 4; *n* = 5); and from pre-infected mice (day 0; *n* = 10). Data are presented as mean ± SD. Multiple comparison of means was performed using Kruskal–Wallis test with Dunn’s post-hoc correction. *p < 0.05. **p < 0.005. ***p < 0.0005. ****p < 0.0001. *ns*, not significant. *Mo-MAC*, monocyte-derived macrophages

The immune cell landscape within the spleen was assessed by flow cytometry at various stages of disease to examine which cell subsets may be driving increased FDG uptake. The total numbers of immune cells in ZIKV spleens, marked as CD45^+^, did not change over time (Fig. 5a). In contrast, the total number of non-immune CD45^–^ cells in ZIKV spleens increased at late disease. However, these cells account for only a small fraction (< 10%) of total cells (CD45^+^ and CD45^–^) in the spleen (Fig. S5a). The CD45^–^ erythrocytes, which may have contributed significantly to the splenomegaly observed in ZIKV-infected mice (Fig. S1c), were not included in this analysis and were lysed during tissue processing. Deeper cell profiling revealed that the subset of CD45^+^ cells most dominant in the spleen are the lymphocytes—mainly the B and T cells—whose absolute cell counts declined in ZIKV infection especially at late disease (Fig. 5c). However, this decline was compensated by concomitant increase in myeloid cell populations infiltrating from the bone marrow, which include granulocytes, monocytes, dendritic cells, and monocyte-derived macrophages (Mo-MAC) (Fig. 5c). The increased demand for immune repopulation of the diseased spleen is being fulfilled by the bone marrow as revealed by the rapid tenfold increase in total bone marrow cell counts most likely contributed by hematopoiesis and proliferation (Fig. S3).

Assessment of the cell proliferation status in ZIKV spleens revealed a peak in CD45^+^ immune cells expressing the proliferation marker ki67 at mid disease. This increase was sixfold higher compared to CD45^–^ cells (Fig. 5a). Further, there was a strong correlation between [^18^F]FDG uptake and proliferating ki67^+^ CD45^+^ spleen immune cells (*ρ* = 0.92; CI: 0.81–0.97; *p* < 0.0001) (Fig. 5b). Within the various immune cell subsets, ki67 expression rapidly increased across all lymphocytes and peaked at mid disease (Fig. 5d), suggesting that these cells are actively replacing lymphocytes that presumably died due to infection. Ki67 expression also significantly increased in infiltrating myeloid cells (Fig. 5d), confirming that the increasing total myeloid cell count during disease was due to both cellular infiltration of actively proliferating cells and in situ proliferation in the spleen (Fig. 5c). Taken together, increased proliferation of both myeloid and lymphoid cells within the ZIKV spleen seem to contribute significantly to [^18^F]FDG uptake.

In contrast to ZIKV, spleens from DENV-infected mice exhibited a rapid decline of CD45^+^ immune cells (Fig. 5a), primarily contributed by loss of both B and T lymphocytes (Fig. S4a). This disappearance was ineffectively compensated by increased infiltration of myeloid cells (Fig. S4b) supplied by the bone marrow (Fig. S3). Increased ki67 expression in immune cells (Fig. 5a), mainly from lymphocytes (Fig. S3b), also failed to counteract the rapid decline of spleen cells in the tissue. Regardless, ki67 expression on immune cells positively correlated with tracer uptake in the spleen (*ρ* = 0.55; CI: 0.17–0.79; *p* = 0.006) (Fig. 5b). Hence, similarly to the observations in ZIKV, immune cell proliferation in the DENV spleen is possibly driving the increased [^18^F]FDG uptake.

## Discussion

To date, ZIKV studies have mainly focused on neurological complications resulting from maternal infection, as well as male infertility post-infection. Hence, the effect of ZIKV infection on the induction of the inflammatory response remains poorly understood. In this preclinical study, we investigated how [^18^F]FDG-PET could serve as a non-invasive imaging biomarker of ZIKV disease and inflammation. The hallmark of murine ZIKV disease in [^18^F]FDG-PET was high tracer uptake in the lymphatic tissues, especially the spleen and axillary lymph nodes (Ax.LN). Consistent with previous studies [24, 25], ZIKV infection in the spleen resulted in gross tissue enlargement and increased viral replication, with the latter trending intimately with tracer tissue uptake. Additionally, this study demonstrated that there are elevated levels of the pro-inflammatory cytokines IL-6 and TNF-α in the spleen, which correlated strongly with tracer in the tissue. In contrast, [^18^F]FDG in Ax.LN only correlated with viral burden and not inflammatory response. With marked differences between [^18^F]FDG uptake and associated ex vivo biomarkers at mid and late disease, early uptake patterns in lymphoid tissues appear to be important in dissecting acute viral ZIKV pathogenesis and host inflammatory response.

The dynamic changes in the immune landscape of ZIKV-infected spleens with increasing disease severity and which immune cell subset/s may be driving the FDG uptake within the tissue have not been studied previously. Here we demonstrate that during ZIKV disease progression, total CD45^+^ immune cells in the spleen were unchanged while total CD45^¯^ non-immune cells increased. Lymphocytes, specifically B and T cells, in the spleen declined during disease, and this is consistent with the known permissiveness of lymphocytes to ZIKV infection [26]. This decline was counterbalanced by increase in myeloid cells (mainly granulocytes, monocytes, monocyte-derived macrophages (Mo-MAC), and dendritic cells) infiltrating the tissue, as supplied by the bone marrow. In addition, immune cell proliferation characterized by increased ki67 expression was found to be significantly elevated in the spleen and strongly correlated with spleen [^18^F]FDG uptake. This implies that the increased energetic demands of highly proliferating cells may be driving the tracer tissue uptake during disease. ki67 expression is reported to positively correlate with [^18^F]FDG uptake in various cancers [27, 28], but this relationship has not been concretely established in the context of infectious disease. A more direct way of resolving which immune cells in the spleen drive [^18^F]FDG uptake is by measuring the glucose transporter expression. The glucose transporter (Glut-3) specifically directs glucose and [^18^F]FDG uptake in immune cells and is further upregulated upon immune-cell activation [29, 30]. Subsequent gamma counting of sorted immune cell subsets gated on high Glut-3 expression collected from animals injected with [^18^F]FDG would then directly identify the specific immune cells driving increased FDG uptake. This method had been described recently in a study aimed at identifying specific cells that take up a radiotracer in the brain [31, 32].

In addition to uptake in lymphoid tissues, we also expected increased [^18^F]FDG uptake in ZIKV brains and gonads—the tissues known from other mouse ZIKV infection models to be highly permissive to infection [20, 24, 33]. PET/CT could not discriminate uptake in the testes from the bladder, despite seeing trends towards elevated tracer uptake in ZIKV-infected testes through DAR and gamma counting. Therefore, [^18^F]FDG may not be a reliable imaging biomarker to interrogate disease and inflammation that had been well characterized in ZIKV-infected gonads [34, 35]. Another known complication of ZIKV disease is neuroinflammation resulting from infection of the central nervous system [33, 36]. Neither PET/CT, DAR, nor gamma counting registered increased [^18^F]FDG uptake in ZIKV brains despite strong evidence of neuroinflammation observed in harvested ZIKV brains at late disease (*data not shown*). The tracer lacks sensitivity in detecting neuroinflammation due to high basal uptake in normal brain tissues. Imaging approaches targeted to molecular markers of neuroinflammation, such as the translocator protein (TSPO), may be more appropriate tools for interrogating ZIKV infection and host response in the brain [37]. In addition, larger preclinical cohort studies are required to confirm the significance of the [^18^F]FDG uptake patterns in brain and testes to show their predictive value.

To date, there are no virus-specific molecular imaging biomarkers. Previous attempts to develop DENV-targeted molecular imaging probes using labeled antisense oligonucleotides that bind to DENV genome sequences resulted in disappointing in vitro results and failed to reach preclinical evaluations [38]. To identify PET imaging biomarkers applicable to various viral diseases, we compared the [^18^F]FDG-PET profile of the ZIKV model with the closely related DENV model using the same type I/II-interferon receptor-deficient AG129 mouse infection model [18, 19]. Both models exhibited similar phenotypes of high lethality (Fig. S1a), severe weight loss (Fig. S1b), and spleen enlargement seen in mid and late ZIKV and in late DENV disease (Fig. S1c). [^18^F]FDG uptake in DENV spleen is not as robust as intestinal FDG uptake as previously reported in preclinical models [10]. However, this current study confirmed that in DENV, spleen [^18^F]FDG uptake is still sufficiently sensitive as a possible imaging biomarker for disease and more closely recapitulates the clinical PET/CT observations in DENV patients [11, 12]. Aside from DENV and ZIKV, lymphoid tissue [^18^F]FDG uptake has also been reported in other viral infections, seen in [^18^F]FDG-PET images of patients infected with chikungunya virus [39], reactivated Epstein-Barr virus [40–42], and reactivated varicella zoster virus or shingles [43]. Lymphoid tissue FDG uptake was also captured in non-human primate models of monkeypox virus infection [44, 45]. Thus, lymphoid tissue [^18^F]FDG uptake may be reflective of the general host immune response to invading viruses, and the dynamic changes in uptake observed in these tissues could be exploited for further development of [^18^F]FDG as an imaging biomarker to support screening of antivirals or vaccine treatments for ZIKV and other viral infections.

In conclusion, [^18^F]FDG uptake in the spleen is a useful surrogate for interrogation of in situ tissue viral burden and inflammation status in this ZIKV murine infection model. Moreover, it can also be used to monitor in situ tissue viral replication and IL-6 expression in the DENV murine model. Studies evaluating these imaging biomarkers in human infections and whether experimental therapeutics that ameliorate disease also dampen [^18^F]FDG uptake in the spleen are also worth pursuing.

## Supplementary Information

Below is the link to the electronic supplementary material.

Supplementary file1 (DOCX 2.02 MB)

## Data Availability

All raw data and materials are available upon request.
