# Supplementary material for: Preclinical evaluation of [18F]FDG-PET as a biomarker of lymphoid tissue disease and inflammation in Zika virus infection
Source: Eur J Nucl Med Mol Imaging. 2022 Jul 25;49(13):4516–28. doi: 10.1007/s00259-022-05892-9 (PMC9309455; doi:10.1007/s00259-022-05892-9)
Supplement: Supplementary file 1 — Supplementary file1 (DOCX 2.02 MB) [file 259_2022_5892_MOESM1_ESM.docx]

**Preclinical evaluation of [^18^F]FDG-PET as a biomarker of lymphoid tissue disease and inflammation in Zika virus infection**

Carla Bianca Luena Victorio^1^, Joanne Ong^1^, Jing Yang Tham^1^, Marie Jennifer Reolo^1^, Wisna Novera^1^,

Rasha Msallam^1^, Satoru Watanabe^2^, Shirin Kalimuddin^2,3^, Jenny G. Low^2,3^, Subhash G. Vasudevan^2^,

Ann-Marie Chacko^1*^

^1^Laboratory for Translational and Molecular Imaging, Cancer and Stem Cell Biology Programme, Duke-NUS Medical School, 8 College Road, Singapore, 169857

^2^Programme in Emerging Infectious Disease, Duke-NUS Medical School, 8 College Road, Singapore, 169857

^3^Department of Infectious Diseases, Singapore General Hospital, 20 College Road, Singapore, 169856

*Corresponding author email: [ann-marie.chacko@duke-nus.edu.sg](mailto:ann-marie.chacko@duke-nus.edu.sg)

Keywords: [^18^F]FDG, Zika, Viral infection, Dengue, AG129 mouse, Viral inflammation

This article is part of the Topical Collection on Infection and inflammation

**Supplemental Files**

**Figure S1**

**
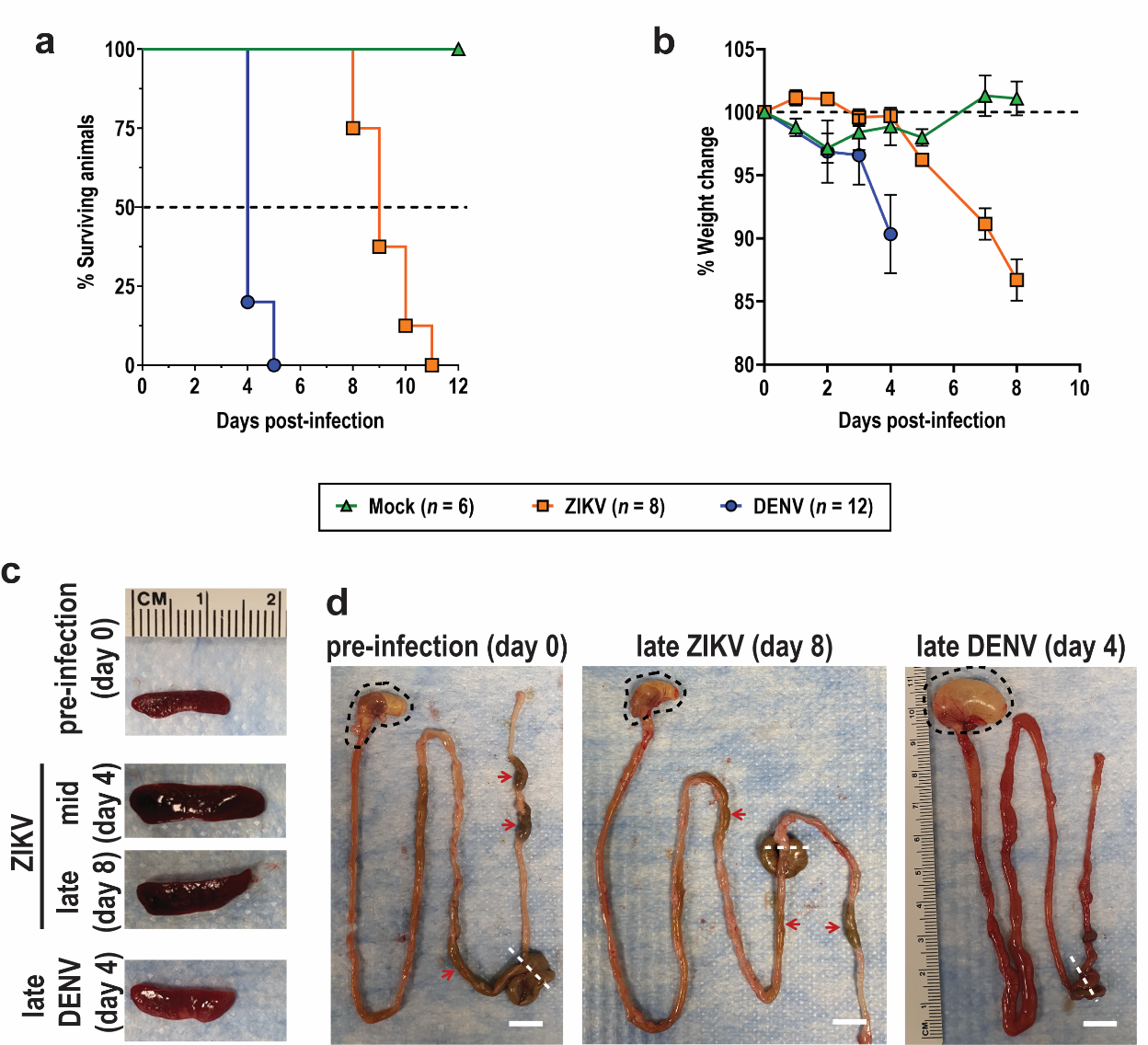
**

**Fig. S1.** Disease kinetics and pathology of acute ZIKV and DENV mouse AG129 disease models. **a** Kaplan-Meier survival and **b** weight loss in lethal Zika virus (ZIKV) infection model with ZIKV^\^ clinical isolate compared to lethal dengue virus (DENV) disease using mouse adapted DENV2 S221 (data shown as mean ± SD). **c-d** Gross pathological changes in the **c** spleen and **d** digestive tract of infected animals. The stomach is highlighted in black dashed lines. Food pellets along the digestive tract are highlighted with red arrowheads. The demarcation between the large intestine and small intestine is indicated by the white dashed lines. *st*, stomach. *S.Int*, small intestine. *L.Int,* large intestine. Scale bar = 1 cm

**Figure S2**

**
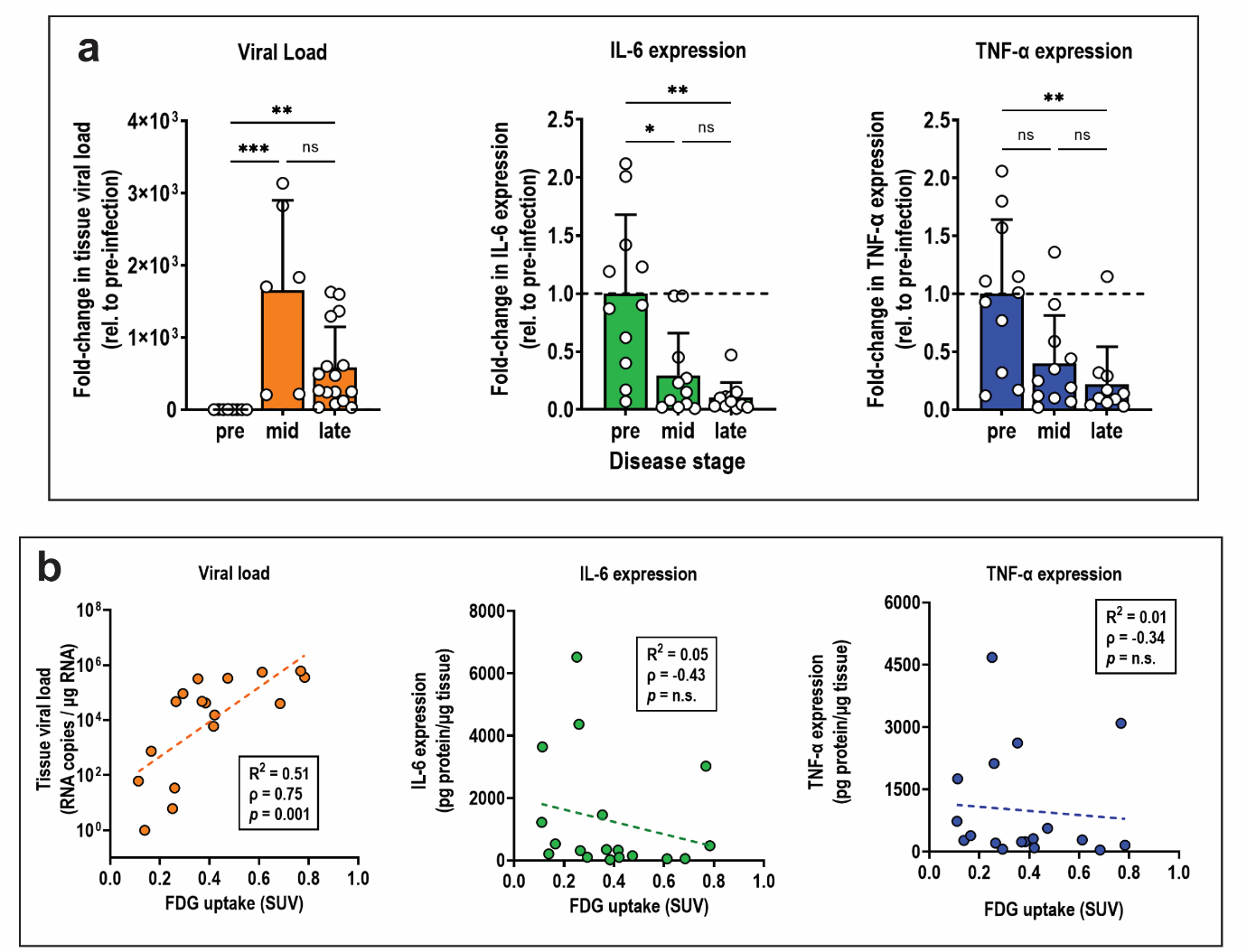
**

**Fig. S2.** Contribution of viral burden and inflammation status on [^18^F]FDG uptake in axillary lymph nodes during acute ZIKV disease. **a** Change in viral load and expression of pro-inflammatory cytokines IL-6 and TNF-α in axillary lymph nodes relative to ZIKV pre-infection. **b** Correlation between axillary lymph node [^18^F]FDG uptake and either tissue viral load, IL-6 expression, or TNF-α expression in ZIKV axillary lymph nodes. Data are presented as mean ± SD. Multiple comparison of means was performed using Kruskal-Wallis test with Dunn’s post-hoc correction. *, *p*<0.05. ** *p*<0.005. ***. *p*<0.0005. *ns*, not significant. Coloured dashed lines in the linear correlation graphs represent the best-fit linear regression trend of the scatter plots. ρ, Spearman correlation coefficient. R^2^, linear regression fitness.

**Figure S3**

**
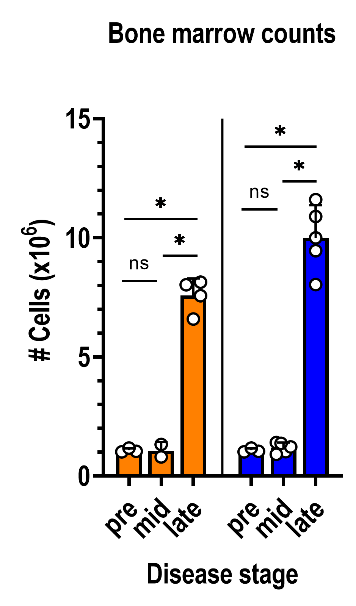
**

**Fig. S3.** Changes in immune cell counts in the bone marrow. Bone marrows from the tibia were harvested at days 0, 4, and 8 post-ZIKV infection and on days 0, 2, and 4 post-DENV infection. These days correspond to pre-infection (pre), mid, and late stages of disease, respectively. Bone marrow-derived immune cells were quantified by flow cytometry. Data are presented as mean ± SD. Multiple comparison of means was performed using Kruskal-Wallis test with Dunn’s post-hoc correction. *, *p*<0.05. *ns*, not significant.

**Figure S4**

**
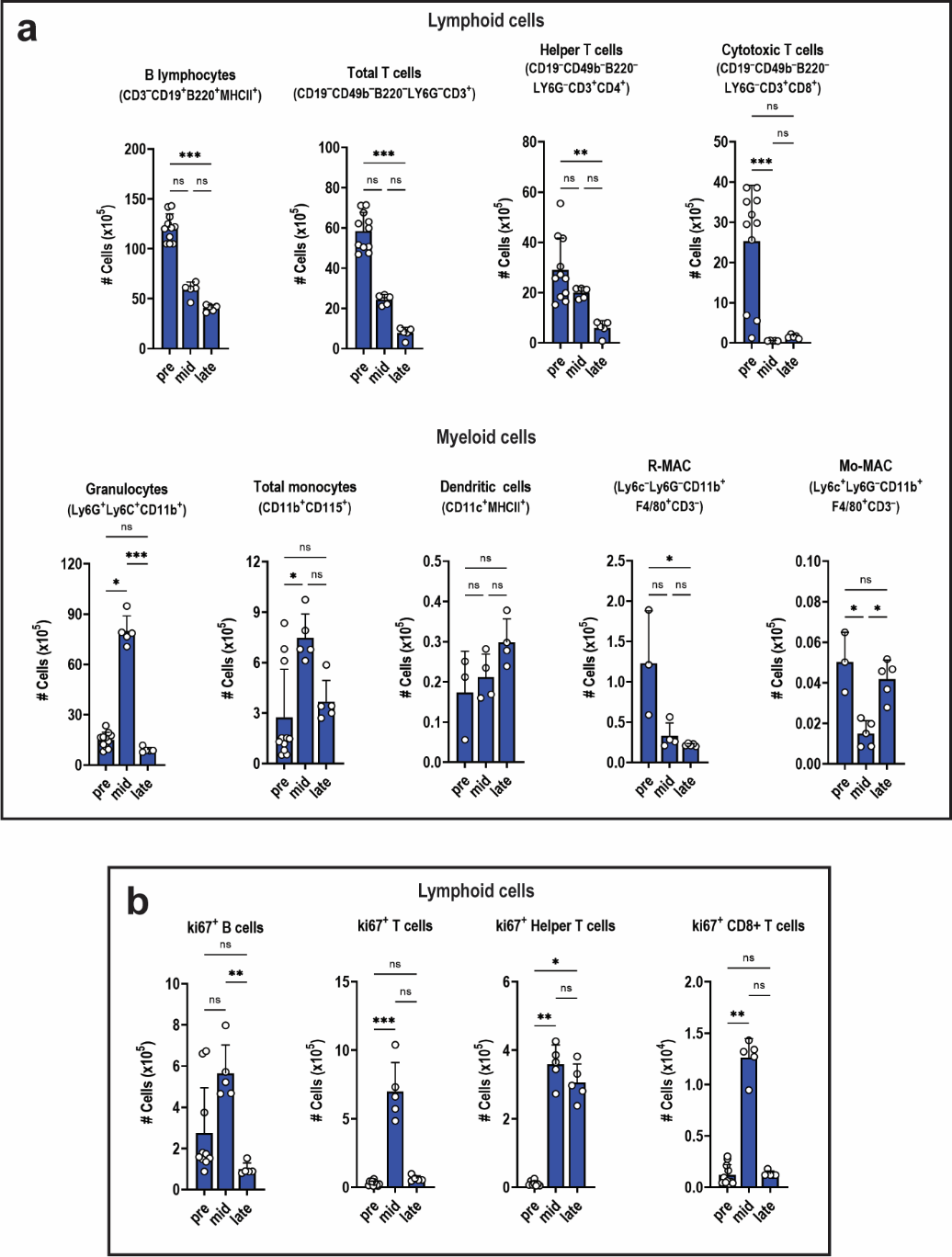
**

**Fig. S4.** Immune profiles and cell proliferation in DENV spleens. **a-b** Quantification of absolute cell numbers of **a** lymphoid and myeloid immune cell subsets, and **b** proliferating (ki67^+^) lymphoid immune cell subsets in DENV spleens harvested at increasing stages of disease severity. Spleens were harvested at days 0, 2, and 4 post-DENV infection, which correspond to pre-infection (pre), mid, and late stages of disease. Data are presented as mean ± SD. Multiple comparison of means was performed using Kruskal-Wallis test with Dunn’s post-hoc correction. *, *p*<0.05. **, *p*<0.005. ***. *p*<0.0005. *ns*, not significant. *R-MAC*, resident macrophages; *Mo-MAC*, monocyte-derived macrophages.

**Table S1.** Comparison of [^18^F]FDG tissue standardized uptake values (SUV) from PET/CT images relative to pre-infection. [^18^F]FDG-PET/CT images of mice infected with either Zika virus (ZIKV) or dengue virus (DENV) were subjected to VOI analysis to determine tracer uptake in various tissues. Data are summarized and presented in **Fig. 2**. Percentage increase in [^18^F]FDG uptake relative to pre-infection (day 0) are shown inside parentheses (%) followed by *p*-values. Means were compared with Kruskal-Wallis test with Dunn’s post-hoc correction. Disease stages are shown as pre-infection (pre; at day 0), mid (day 4 in ZIKV and day 2 in DENV), and late (day 8 in ZIKV and day 4 in DENV). *Ax.LN*, axillary lymph node; *In.LN,* inguinal lymph node; *Cer.LN*, cervical lymph node. *ns*, not significant.

| [^18^F]FDG SUV ± SD (% change relative to pre-infection; *p*-value) | | | | | | | | |
| --- | --- | --- | --- | --- | --- | --- | --- | --- |
| Disease stage |  | **pre** |  | **ZIKV** | |  | **DENV** | |
|  |  |  |  | **mid** | **late** |  | **mid** | **late** |
| Gut |  | 1.62 ± 0.46 |  | 1.67 ± 0.52  (3.3%; *n.s.*) | 1.71 ± 0.53  (6.0%; *n.s.*) |  | 1.51 ± 0.16  (-6.7%; *n.s.*) | 2.41 ± 0.52  (49.2%; *0.011*) |
| Spleen |  | 0.94 ± 0.26 |  | 1.61 ± 0.62  (71.9%; *0.004*) | 1.10 ± 0.46  (17.4%; *n.s.*) |  | 1.53 ± 0.24  (63.2%; *0.001*) | 1.43 ± 0.17  (53.2%; *0.002*) |
| Ax.LN |  | 0.44 ± 0.39 |  | 0.83 ± 0.40  (89.8%; *0.0005*) | 0.38 ± 0.14  (-13.2%; *n.s.*) |  | 0.95 ± 0.45  (118%; *0.002*) | 0.65 ± 0.42  (50.4%; *n.s.*) |
| Cer.LN |  | 0.53 ± 0.33 |  | 0.92 ± 0.41  (74.9%; *0.004*) | 0.70 ± 0.36  (32.5%; *n.s.*) |  | 0.62 ± 0.17  (17.6%; *n.s.*) | 0.36 ± 0.18  (-32.2%; *n.s.*) |
| In.LN |  | 0.27 ± 0.31 |  | 0.52 ± 0.44  (94.7%; *n.s.*) | 0.90 ± 1.34  (237.9%; *n.s.*) |  | 0.59 ± 0.42  (121.3%; *n.s.*) | 1.05 ± 0.66  (295.1%; <*0.0001*) |

**Table S2.** *Ex vivo* [^18^F]FDG biodistribution studies. [^18^F]FDG tissue biodistribution represented as mean % injected dose (% ID) and % change in uptake relative to pre-infected controls (Pre). *s.intestine*, small intestines; *l.intestine*, large intestines; *Ax.LN*, axillary lymph node; *In.LN,* inguinal lymph node; *Cer.LN*, cervical lymph node. *ns*, not significant.

| Tissue |  | Pre |  | Late ZIKV (day 8) | | |  | Late DENV (day 4) | | |
| --- | --- | --- | --- | --- | --- | --- | --- | --- | --- | --- |
|  |  | **[^18^F]FDG uptake**  **(% ID ± SD)** |  | **[^18^F]FDG uptake**  **(% ID ± SD)** | **%change**  ***vs.* pre** | ***p*-value** |  | **[^18^F]FDG uptake**  **(% ID ± SD)** | **%change**  ***vs.* pre** | ***p*-value** |
| blood |  | 0.87 ± 0.18 |  | 0.70 ± 0.15 | -20 % | *n.s.* |  | 0.62 ± 0.33 | -29% | *n.s.* |
| heart |  | 1.67 ± 1.74 |  | 2.87 ± 0.53 | 72 % | *n.s.* |  | 0.64 ± 0.37 | -61 % | *n.s.* |
| lungs |  | 0.52 ± 0.03 |  | 0.57 ± 0.04 | 9 % | *n.s.* |  | 0.96 ± 0.32 | 83 % | 0.0006 |
| kidneys |  | 1.99 ± 0.39 |  | 2.16 ± 0.96 | 8 % | *n.s.* |  | 2.15 ± 0.57 | 8% | *n.s.* |
| brain |  | 4.76 ± 1.49 |  | 4.93 ± 0.42 | 3 % | *n.s.* |  | 6.43 ± 1.43 | 35 % | *n.s.* |
| spleen |  | 0.16 ± 0.03 |  | 1.54 ± 0.25 | 879 % | *0.0001* |  | 2.37 ± 0.58 | 1,408 % | 0.0007 |
| pancreas |  | 0.30 ± 0.17 |  | 0.48 ± 0.47 | 59 % | *n.s.* |  | 0.25 ± 0.12 | -16 % | *n.s.* |
| liver |  | 1.06 ± 0.21 |  | 1.25 ± 0.18 | 17 % | *n.s.* |  | 2.22 ± 0.76 | 109 % | 0.002 |
| stomach |  | 0.54 ± 0.18 |  | 0.64 ± 0.04 | 17 % | *n.s.* |  | 1.92 ± 0.38 | 253 % | 0.0013 |
| s. intestine |  | 2.35 ± 0.59 |  | 2.54 ± 0.32 | 8 % | *n.s.* |  | 27.8 ± 6.78 | 1,084 % | 0.0032 |
| l. intestine |  | 1.86 ± 0.24 |  | 2.12 ± 0.09 | 14 % | *n.s.* |  | 9.08 ± 4.17 | 389 % | 0.0008 |
| testes |  | 1.12 ± 0.22 |  | 1.39 ± 0.25 | 24 % | *n.s.* |  | 1.09 ± 0.33 | -3 % | *n.s.* |
| muscle |  | 0.18 ± 0.01 |  | 0.19 ± 0.13 | 3 % | *n.s.* |  | 0.11 ± 0.09 | -40 % | *n.s.* |
| bone |  | 0.10 ± 0.05 |  | 0.14 ± 0.03 | 46 % | *n.s.* |  | 0.25 ± 0.10 | 164 % | 0.01 |
| Ax.LN |  | 0.02 ± 0.01 |  | 0.07 ± 0.03 | 305 % | 0.03 |  | 0.08 ± 0.02 | 306 % | 0.005 |
| Cer.LN |  | 0.02 ± 0.01 |  | 0.11 ± 0.05 | 558 % | 0.04 |  | 0.13 ± 0.05 | 723 % | 0.004 |
| In.LN |  | 0.01 ± 0.00 |  | 0.04 ± 0.02 | 366 % | 0.048 |  | 0.05 ± 0.03 | 446 % | 0.023 |
